# Av*β*3 Single-Stranded DNA Aptamer Attenuates Vascular Smooth Muscle Cell Proliferation and Migration via Ras-PI3K/MAPK Pathway

**DOI:** 10.1155/2020/6869856

**Published:** 2020-01-16

**Authors:** Hong-Bing Wu, Zhi-Wei Wang, Feng Shi, Zong-Li Ren, Luo-Cheng Li, Xiao-Ping Hu, Rui Hu, Bo-Wen Li

**Affiliations:** Department of Cardiovascular Surgery, Renmin Hospital of Wuhan University, Wuhan, China

## Abstract

**Objectives:**

To observe the effect of av*β*3 single-stranded (ss) DNA on proliferation and migration of vascular smooth muscle cells (VSMCs) and its potential mechanism.

**Background:**

Percutaneous transluminal coronary angioplasty (PTCA) is currently the preferred method for the treatment of coronary heart disease. However, vascular restenosis still occurs after PTCA treatment, severely affecting the clinical efficacy of PTCA. Integrin av*β*3, which is widely expressed on various cell surfaces, plays an important role in the proliferation and migration of VSMCs.

**Methods:**

In this experiment, we used systematic evolution of ligands by exponential enrichment (SELEX) to screen out av*β*3 ssDNA, which has high affinity and specificity to the av*β*3 protein. MTT, Transwell, and cell scratch assays were carried out to examine the effect of av*β*3 ssDNA on the proliferation and migration of VSMCs. Flow cytometry was performed to detect apoptosis and cell cycle progression. The effect of av*β*3 ssDNA on the Ras-phosphatidylinositol-4,5-bisphosphate 3-kinase/mitogen-activated protein kinase (PI3K/MAPK) signaling pathway was evaluated by quantitative reverse transcription polymerase chain reaction and western blot.

**Results:**

In the present study, we found that av*β*3 ssDNA significantly decreased the expression of osteopontin, focal adhesion kinase, Ras, p-PI3K, and p-MAPK at both mRNA and protein levels (*P* < 0.05). Av*β*3 ssDNA also inhibited VSMC proliferation and migration while promoting apoptosis (*P* < 0.05), as demonstrated by the upregulation of the proapoptotic proteins Bax and active caspase 3 (*P* < 0.05).

**Conclusions:**

The findings suggest that av*β*3 ssDNA inhibited the proliferation and migration of VSMCs by suppressing the activation of Ras-PI3K/MAPK signaling.

## 1. Introduction

Coronary heart disease is a high-risk condition among cardiovascular diseases and has one of the highest mortality rates worldwide. It is reported that cardiovascular diseases accounted for greater than 40% of disease-related deaths among residents in 2017 in China [1], which was much higher than the incidence of tumors and other diseases. Coronary heart disease is characterized by myocardial ischemia, hypoxia, and necrosis caused by coronary artery stenosis and obstruction. Percutaneous transluminal coronary angioplasty (PTCA) is one of the main methods for the clinical treatment of coronary heart disease, but vascular restenosis still occurs after treatment, severely limiting the efficacy of PTCA in clinical practice. The proliferation and migration of vascular smooth muscle cells (VSMCs) has been considered the major cause of post-PTCA restenosis [2, 3]. Therefore, the development of ways to inhibit VSMC migration and proliferation has become an urgent problem in cardiovascular research.

Integrin av*β*3, which binds to extracellular matrix proteins as a ligand, is widely expressed on the surface of endothelial cells, platelets, monocytes, and other cells. It is closely related to embryonic development, angiogenesis, invasion and metastasis of tumor cells, and inflammation [4, 5]. Av*β*3 is highly expressed in osteoclasts and is the most important integrin regulating osteoclast function, participating in osteoclast differentiation, migration, adhesion, and formation of closed regions [6]. Contrarily, av*β*3 is hardly expressed in mature vascular endothelial cells but is highly expressed in activated vascular endothelial cells. It mediates the proliferation, migration, and adhesion of vascular endothelial cells, transmits survival signals into cells, and inhibits apoptosis [7].

As a star molecule in targeted therapy, nucleic acid aptamer drugs have been used for many clinical applications, including cancer [8, 9], neovascularization [10], and acute coronary syndrome [11]. Aptamers specifically bind to the target protein to exert their therapeutic effect. Studies have shown that aptamers restricted cell proliferation and migration by inhibiting translation and blocking cell adhesion [12, 13]. Cerchia observed that aptamers not only bind to target proteins but also block downstream signaling of the target protein, resulting in cellular and molecular changes [14]. Kimoto et al. found that Raf-1 aptamers can bind to the Ras domain of the Raf-1 protein, making it unable to be activated by Ras for signal transduction [15]. Although there is an increasing number of studies on aptamers, as far as we know, few studies have been carried out to develop a specific aptamer that binds directly to the av*β*3 protein.

We hypothesized that blockage of av*β*3 protein activation may be a therapeutic target to treat post-PTCA vascular restenosis. In this study, we screened single-stranded (ssDNA) aptamers with high affinity and specificity for the av*β*3 protein using systematic evolution of ligands by exponential enrichment (SELEX) and studied the effects and specific mechanism of av*β*3 on the proliferation, migration, and apoptosis of VSMCs.

## 2. Materials and Methods

### 2.1. Cell Culture

Ten Sprague-Dawley rats (specific-pathogen-free, 250–270 g) were provided by the Hubei Province Disease Control Center (NO. 211002300042744). After anesthesia, the aorta thoracica and aorta ventralis were extracted, the endangium and adventitia were removed, and the intermediate layer of vascular tissue was placed into precooled phosphate-buffered saline to wash away the residual blood. The tissue was cut into 1-mm^3^ pieces and incubated in a T25 flask at 37°C in an atmosphere containing 5% CO_2_ for 6 hours with 2.5 ml of culture solution containing 97% smooth muscle cell medium (SMCM, 1101, ScienCell, USA), 2% fetal bovine serum (FBS), and 1% smooth muscle cell growth supplement. The medium was replaced every 72 hours. All protocols and procedures were in accordance with the National Institutes of Health Guide for the Care and Use of Laboratory Animals and were approved by the Animal Care and Use Committee, Wuhan Myhalic Biotechnology Co. Ltd. (HLK-20180920-01).

To identify the isolated VSMCs, the shapes of the extracted cells were analyzed. The cells were dyed for *α*-smooth muscle actin (*α*-SMA) and smooth muscle protein 22*α* (SM22*α*) and observed by using a fluorescence microscope.

### 2.2. In Vitro Selection of av*β*3 ssDNA Aptamers

According to previously reported methods [16, 17], we established a random oligonucleotide library and selected av*β*3 ssDNA aptamers (from humans, unlabeled) in vitro. The random oligonucleotide library sequence was CCCGCTGTAACCTGAAA-N40-AGTTCCCAGTCAGCGAG and the av*β*3 sequences were 5′-CCCGCTGTAACCTGAAA-3′ (forward) and 5′-CTCGCTGACTGGGAACT-3′ (reverse). Double-stranded DNA was obtained using ssDNA as a template through symmetric polymerase chain reaction (PCR) amplification, and the obtained double-stranded DNA was used as a template to obtain ssDNA by asymmetric PCR amplification. The PCR amplification reaction system is shown in Table 1. The PCR conditions were as follows: denaturation at 94°C for 3 min, followed by 35 cycles of denaturation at 94°C for 40 s, annealing at *X*°C (*X* stands for 53, 53.8, 55.3, 57.6, 60.3, 62.6, 64.1, and 65) for 30 s, and extension at 72°C for 45 s. The PCR products were identified by 4% agarose gel electrophoresis. Subsequently, the affinity and specificity of each round of ssDNA were identified by the ELISA assay until the binding rate and affinity did not increase significantly.

Suitable aptamers were screened out from the 12th round of SELEX and transformed into *Escherichia coli* DH5*α* strains after purification. Plasmid DNA was isolated from individual clones, sequenced, and analyzed. The secondary structure of the aptamers was analyzed using Dnaman 5.29 software (Lynnon Corp., Quebec, QC, Canada).

### 2.3. Co-Immunoprecipitation

Samples were collected, and an appropriate amount of precooled cell lysis buffer was added. After 30 minutes of lysis on ice, centrifugation was carried out for 30 minutes, and 1 *μ*g of integrin av*β*3 antibody was added to each sample and incubated overnight at 4°C. 10 ml of protein A agarose beads were washed with pyrolysis buffer three times and centrifuged with 3000 rpm three times for 3 min each. Then, the protein A agarose beads were mixed with the samples, cultured at 4°C for 4 h, and centrifuged at 3000 rpm for 3 min. The supernatant was discarded, and the agarose beads were washed 3 times with 1 ml of pyrolysis buffer. Finally, 15 *μ*l of 2x sodium dodecyl sulfate sample buffer was added, and the samples were analyzed by western blot.

### 2.4. Transfection of av*β*3 Protein

Av*β*3 protein was transfected according to the method of Mou et al. [18]. We designed the primers of *β*3 by referring to the full-length cDNA sequence of human integrin *β*3 published by GenBank: P1, 5′-ATTATTCTACGGAC-GAGATGCGAGC-3′; P4, 5′-GGGCTCGAGTTATA-CAGTGGG-TTGTT-3′. Then, they were transfected into pBluescript II SK (+) Integrin *β*3 (KL1892, KALANG, China) plasmid. Thereafter, the open reading frame of the *β*3 subunit amplified from pBluescript II SK (+) Integrin *β*3 plasmid was transfected into the eukaryotic expression vector pcDNA 3.1/V5-His-TOPO (K480001, Thermo Fisher, USA) to construct pcDNA3.1-*β*3 vectors. The pcDNA3.1-*β*3 vectors were then cotransfected into the VSMCs with pcDNA3-av vectors (13032, Addgene, USA) according to the instructions of the Lipofectamine 2000 kit (11668-027, Invitrogen, USA). VSMCs were randomly divided into six groups: VSMC (no treatment), VSMC + platelet-derived growth factor (PDGF) 4 ng/ml, VSMC + PDGF + av*β*3 ssDNA 20 *μ*g/ml, VSMC + PDGF + av*β*3 ssDNA 40 *μ*g/ml, VSMC + PDGF + av*β*3 ssDNA 80 *μ*g/ml, and VSMC + PDGF + cilengitide (positive control).

### 2.5. 3-(4,5-Dimethylthiazol-2-yl)-2,5-diphenyltetrazolium Bromide (MTT) Assay

The proliferation of VSMCs was detected using the MTT kit (PAB180013, Bioswamp) according to the manufacturer's instructions. We collected cells in the logarithmic growth phase, adjusted the cell concentration to 5 × 10^3^ cells/well in a 96-well plate, and added 20 *μ*l of MTT solution to each well. The cells were incubated at 37°C in a 5% CO_2_ incubator overnight, and 150 *μ*l of dimethyl sulfoxide (D2650, Sigma) was added to each well. The optical density was measured at 490 nm with a microplate reader (Multiskan FC, Thermo).

### 2.6. Transwell Assay

The cells were cultured in serum-free SMCM 24 h before the experiment and resuspended in SMCM containing 1% FBS at 1 × 10^5^ cells/ml. Then, the cells were inoculated into Transwell chambers, and 0.75 ml of SMCM containing 10% FBS was added into the lower 24-well plate. The cells were cultured in 5% CO_2_ at 37°C for 48 h. Then, 1 ml of 4% formaldehyde solution was added into each well, and the cells were placed at room temperature for 10 minutes for immobilization. After dyeing with 0.5% crystal violet solution (PAB180004, Bioswamp) for 30 minutes, the cells were observed under a microscope.

### 2.7. Flow Cytometry

Apoptosis and cell cycle progression of VSMCs were analyzed using flow cytometry according to the manufacturer's instructions. The contents of cells at every stage of the cell cycle and the percentage of apoptosis were measured, and the data were analyzed using CytExpert software.

### 2.8. Western Blot

Proteins were extracted from cells, and their concentration was measured using a bicinchoninic acid protein assay kit (Beyotime, China). Total protein was separated by 8% or 12% sodium dodecyl sulfate-polyacrylamide gel electrophoresis and transferred to polyvinylidene fluoride membranes. The following primary antibodies were used: osteopontin (OPN, 1 : 1000, PB0589, Boster), focal adhesion kinase (FAK, 1 : 500, BM4303, Boster), p-FAK (1 : 500, BM4426, Boster), Ras (1 : 400, BM4281, Boster), mitogen-activated protein kinase (MAPK, 1 : 1000, BM4439, Boster), p-MAPK (1 : 2000, P00176, Boster), phosphatidylinositol 3-kinase (PI3K, 1 : 1000, BM5187, Boster), p-PI3K (1 : 1000, ab182651, Abcam), and GAPDH (1 : 1000, PAB36264, Bioswamp). After three washes with phosphate-buffered saline/Tween 20, the membranes were incubated with horseradish peroxidase-conjugated secondary goat anti-rabbit IgG (1 : 20000, PAB160011, Bioswamp). Protein bands were visualized by enhanced chemiluminescence color detection (Tanon-5200, TANON) and analyzed using AlphaEase FC gel image analysis software.

### 2.9. Quantitative Reverse Transcription PCR (qRT-PCR)

Whole RNA was extracted from cell samples using Trizol reagent according to the manufacturer's procedures, and cDNA was synthesized using a reverse transcriptase kit (TAKARA, USA). qRT-PCR was performed using a real-time system (Bio-Rad) using the SYBR Green PCR Kit (KM4101, KAPA Biosystems). Each reaction was performed in duplicate, and the results were analyzed by the 2^−△△Ct^ method. The primers were designed and configured by Nanjing Kingsley Biotechnology Co., Ltd. (Table 2).

### 2.10. Statistical Analysis

All data are presented as mean ± standard deviation. The SPSS 19.0 software was used for statistical analyses, and GraphPad Prism 5.0 was used to prepare the figures. The data were evaluated for statistical significance using one-way analysis of variance. Statistical significance was established at *P* < 0.05.

## 3. Results

### 3.1. Isolation of High-Affinity av*β*3 Protein Aptamers

**Figure 1:**
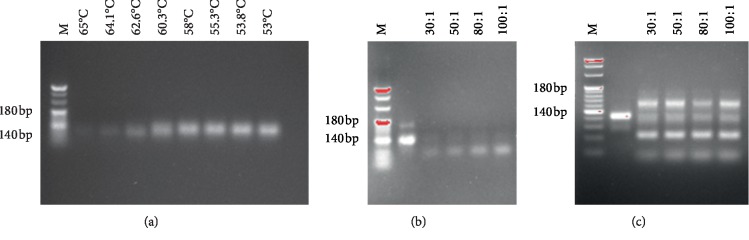
4% agarose gel electrophoresis under various conditions: (a) direct and (b) indirect asymmetric PCR; (c) indirect asymmetric PCR amplification of large amounts of ssDNA after optimization of PCR conditions.

**Figure 2:**
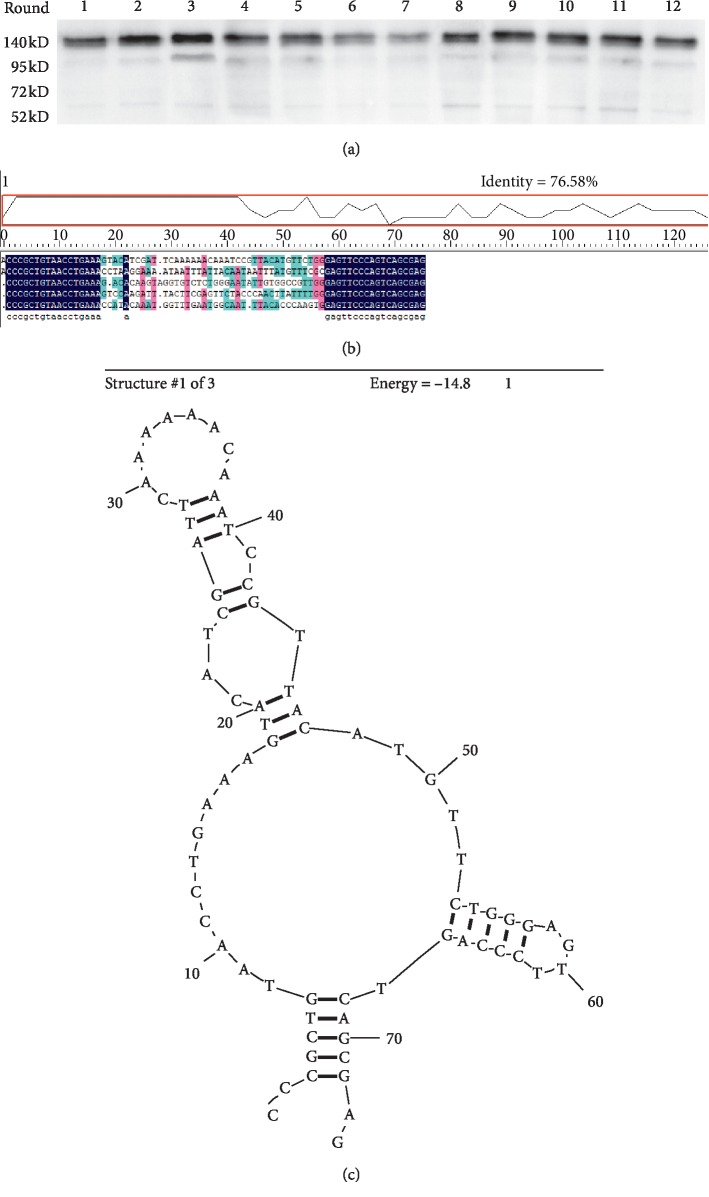
Screening, sequencing, and prediction of av*β*3 ssDNA: (a) expression of av*β*3 protein was observed by immunoprecipitation; (b) cloning and sequencing results; (c) secondary structure of av*β*3 ssDNA for subsequent experiments.

In this study, av*β*3 protein was selected as the target and a random ssDNA library was used as the screening ligand. After 12 rounds of screening, different aptamers were obtained using SELEX. As shown in Figures 1(a)–1(c), the optimum annealing temperature for the symmetric PCR reaction was 58°C. Indirect asymmetric PCR is feasible for the preparation of large amounts of ssDNA, and the primer dilution ratio is 100 : 1. We then determined the binding rate and affinity of av*β*3 ssDNA to the av*β*3 protein in each round by ELISA. The results showed that, at the 11th round, the ssDNA had the highest binding rate and affinity to av*β*3 (Tables 3 and 4). We further performed immunoprecipitation on the av*β*3 protein and found that the bands appeared in the expected position, which confirmed the accuracy of the screening (Figure 2(a)).

Five aptamer individual clones have obtained and sequenced, and the secondary structure of all adapters was predicted by using DNAMAN 5.29 software (Lynnon Corp.) (Figure 2(b)). Secondary structure of the adapter for subsequent experiments was shown in Figure 2(c), and the secondary structure of the remaining four adapters was provided as Supplementary materials ([Supplementary-material supplementary-material-1]). The results showed that the binding sites of the av*β*3 protein may be located at the end of a stem-loop structure (Figures 2(b) and 2(c)).

### 3.2. Isolation and Identification of VSMCs

**Figure 3:**
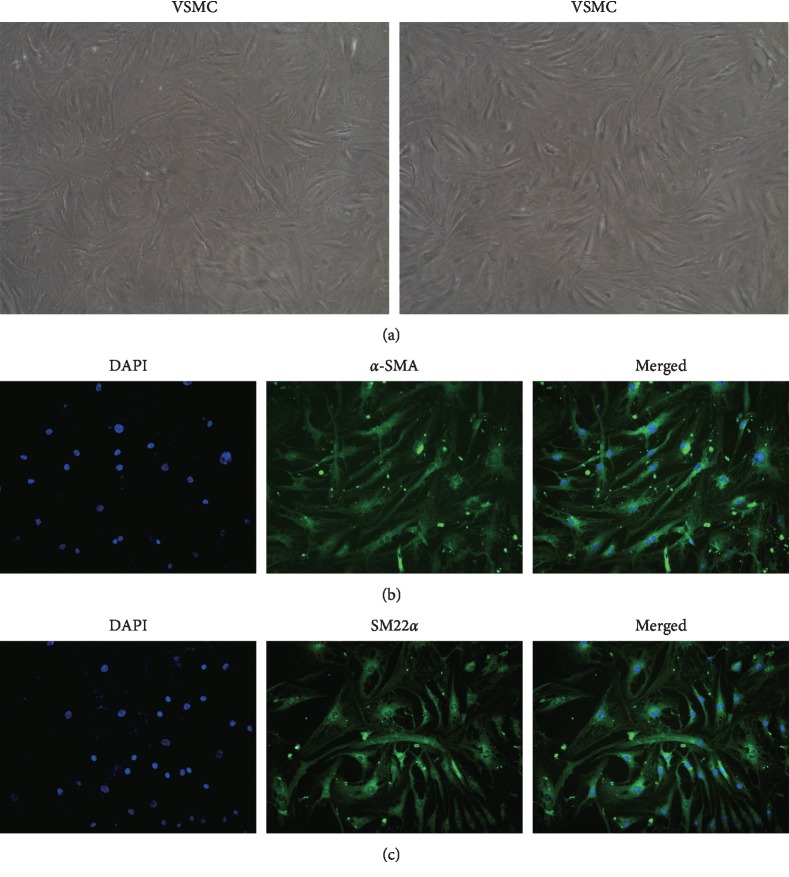
Identification of VSMCs. Scale bar = 100 *μ*m. (a) Bright-field microscopy of VSMCs. Identification of VSMCs by immunofluorescence staining with (b) *ɑ*-SMA and (c) SM22*ɑ*. Scale bar = 100 *μ*m.

To identify primary VSMCs, we showed that the adherent VSMCs were grown at a high density, and the cells were arranged in a spindle or bundle shape (Figure 3(a)). We also assessed the purity of primary VSMCs by detecting the green fluorescence of *α*-SMA and SM22*α* using a fluorescence microscope (200x). The results in Figures 3(b) and 3(c) showed that the isolated cells were VSMCs and can be used in subsequent experiments.

### 3.3. av*β*3 ssDNA Inhibited VSMC Proliferation and Migration

**Figure 4:**
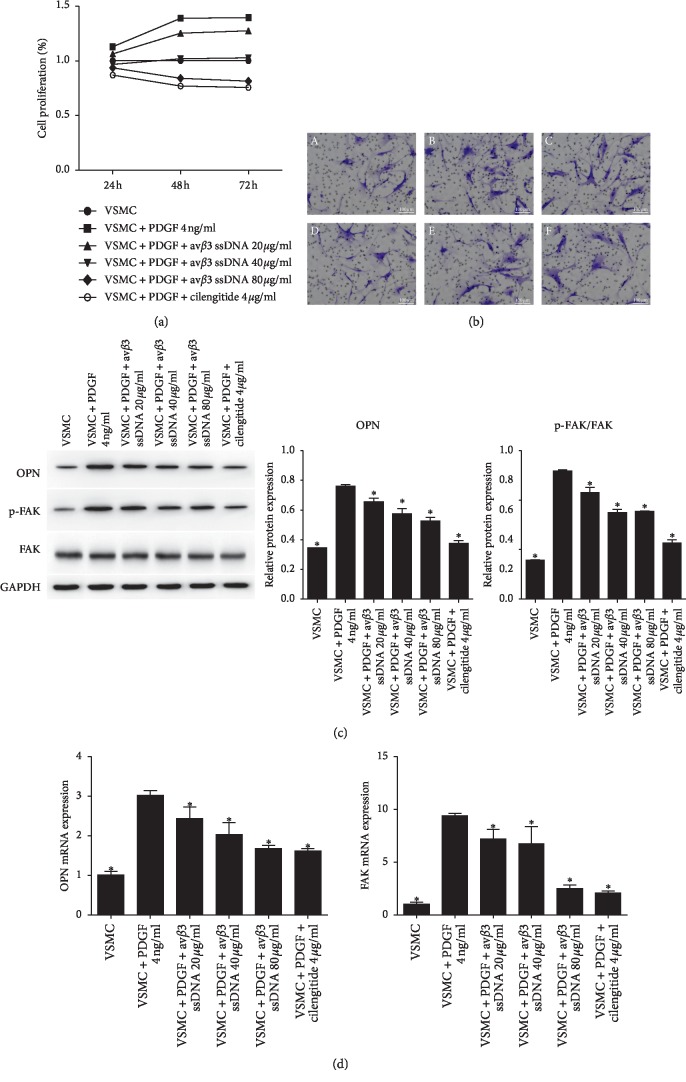
Effect of av*β*3 ssDNA on VSMC proliferation and migration. (a) Cell proliferation measured by MTT. (b) Cell migration measured by the Transwell assay. Scale bar = 100 *μ*m. (c) Detection and quantification of OPN and p-FAK/FAK protein expression. (d) mRNA expression of OPN and FAK. The results are presented as the mean ± SD, *n* = 3. ^*∗*^*P* < 0.05 vs. VSMC + PDGF, 4 ng/ml.

The addition of PDGF significantly increased the proliferation of VSMCs. Compared with the VSMC + PDGF group, the cell proliferation of VSMCs treated with av*β*3 ssDNA decreased in a concentration-dependent manner (Figure 4(a)). To detect the effect of av*β*3 ssDNA on cell migration, we carried out a Transwell assay (Figure 4(b)). Compared with the VSMC + PDGF group, the addition of cilengitide or av*β*3 ssDNA (20 *μ*g/ml, 40 *μ*g/ml, or 80 *μ*g/ml) decreased VSMC migration, and the decrease was positively correlated with the concentration of av*β*3 ssDNA. We further investigated the expression of OPN and FAK, which are related to VSMC proliferation and migration. The relative protein (Figure 4(c)) and mRNA (Figure 4(d)) expression of OPN and p-FAK/FAK in VSMCs treated with PDGF and av*β*3 ssDNA were significantly lower than those in VSMC treated with PDGF only (*P* < 0.05).

### 3.4. av*β*3 ssDNA Promoted VSMC Apoptosis

**Figure 5:**
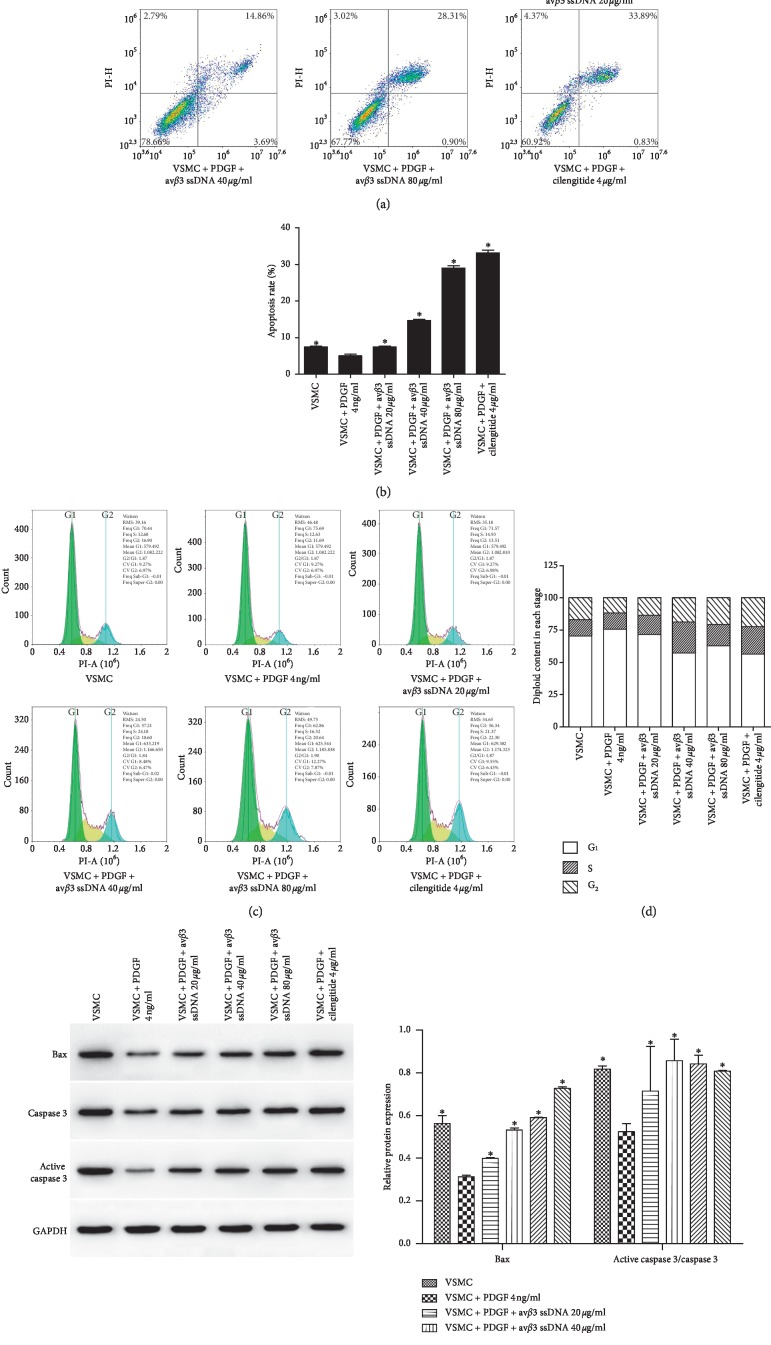
The impact of av*β*3 ssDNA on VSMC apoptosis and cell cycle progression. (a) Detection and (b) quantification of VSMC apoptosis. (c) Detection and (d) quantification of cell cycle progression in VSMCs. (e) Protein expression of Bax and active caspase 3/caspase 3. The results are presented as the mean ± SD, *n* = 3. ^*∗*^*P* < 0.05 vs. VSMC + PDGF 4 ng/ml.

Flow cytometry was used to detect the effect of av*β*3 ssDNA on VSMC apoptosis. The results in Figures 5(a) and 5(b) revealed that av*β*3 ssDNA increased the degree of apoptosis compared to that in VSMCs treated with PDGF only (*P* < 0.05). With the increase in av*β*3 ssDNA concentration, cell apoptosis increased gradually. Figures 5(c) and 5(d) indicated that av*β*3 ssDNA significantly increased the proportion of cells in the S and G2 phase compared to PDGF treatment only. We further measured the apoptosis-related proteins Bax and active caspase 3 (Figure 5(e)) and demonstrated that their content in the av*β*3 ssDNA groups was much higher than that in the VSMC + PDGF group (*P* < 0.05).

### 3.5. Effect of av*β*3 ssDNA on the Ras-PI3K/MAPK Pathway in VSMCs

**Figure 6:**
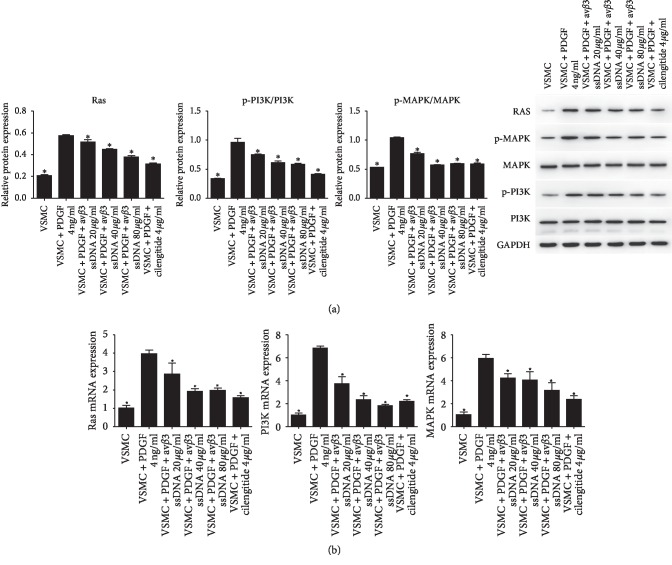
Effect of av*β*3 ssDNA on the protein and mRNA expression of Ras/PI3K/MAPK. (a) Western blot analysis of the protein expression of Ras, p-PI3K, and p-MAPK. (b) qRT-PCR analysis of the mRNA expression of Ras, PI3K, and MAPK. The results are presented as the mean ± SD, *n* = 3. ^*∗*^*P* < 0.05 vs. VSMC + PDGF 4 ng/ml.

To investigate the influence of av*β*3 ssDNA on Ras-PI3K-MAPK signaling in VSMCs, we measured the expressions of Ras, p-MAPK, and p-PI3K using qRT-PCR and western blot (Figures 6(a) and 6(b)). We observed that compared with that, in the VSMC + PDGF group, the protein expression of Ras, p-MAPK/MAPK, and p-PI3K/PI3K and the mRNA expression of Ras, MAPK, and PI3K were significantly decreased by av*β*3 ssDNA at all concentrations (*P* < 0.05). Av*β*3 ssDNA at 80 *μ*g/ml had the greatest effect among all av*β*3 ssDNA groups.

## 4. Discussion

Studies have shown that the proliferation and migration of VSMCs are directly proportional to the degree of vascular injury [19]. Therefore, inhibiting the proliferation and migration of VSMCs may play a therapeutic role in restenosis after PTCA. Integrins are an important component of cell adhesion that mainly mediate adhesion and bidirectional signal transduction between cells and the extracellular matrix. Integrin av*β*3 can be expressed in a variety of cells and has important functions in cell proliferation, migration, invasion, inflammation, and wound healing. Studies have shown that av*β*3 plays a critical role in a variety of diseases. For example, Camorani et al. observed that aptamer-mediated impairment of the epidermal growth factor receptor-integrin *α*v*β*3 complex inhibited vasculogenic mimicry and growth of triple-negative breast cancers [20]. Su et al. revealed that av*β*3 promoted the proliferation and differentiation of osteoblasts by activating the extracellular signal-regulated kinase (Erk) pathway to treat osteoporosis [21]. In addition, av*β*3 functions in myocardial fibrosis via the TGF*β* signaling pathway, demonstrating its implications in various diseases via multiple pathways. Graf et al. observed that av*β*3 participated in the regulation of vascular remodeling processes and the formation of atherosclerosis [22]. Sajid et al. used av*β*3 inhibitors to intervene in eight animal models of vascular injury and found that inhibition of av*β*3 activity suppressed VSMC proliferation and migration [23]. Our previous study indicated that Ras ssDNA aptamers inhibited VSMC proliferation and migration through the MAPK and PI3K pathways [10], and Illario et al. found that av*β*3 mediated the proliferation of fibronectin-induced thyroid cells via Ras/Raf/Mek/Erk and Ca2+/CaMKII signaling [24]. These findings collectively indicate that inhibiting the activity of the av*β*3 protein could reduce the proliferation and migration of VSMCs.

The findings of this study showed that the effect of av*β*3 ssDNA on VSMC proliferation and migration was directly proportional to its concentration. Among proteins that influence cell proliferation and migration [25–27], OPN is widely distributed in various tissues and cells, facilitating cell adhesion by binding to multiple integrin receptors on the cell surface by relying on RGD sequences (*α*v*β*1, *α*v*β*3, *α*v*β*5, *α*v*β*1, and *α*8*β*1) and independent RGD sequences (*α*4*β*1 and *α*9*β*1). OPN can promote the migration of various cells such as osteoclasts, fibroblasts, and smooth muscle cells [28] and is the main regulator of adhesion in blood vessels. Zhang et al. found that berberine inhibited the calcification of VSMCs by inhibiting the expression of OPN [29]. FAK is an important member of the integrin-mediated signal transduction pathway that affects cell adhesion, proliferation, and migration. Inhibition of FAK activity can significantly impair cell proliferation and promote apoptosis [30]. Xie et al. found that graphene-induced bone formation by promoting the expression of OPN, FAK, and other proteins [31]. We further detected changes in the expression levels of OPN and p-FAK and found that they were downregulated with the increase in av*β*3 ssDNA concentration. Flow cytometry showed that av*β*3 ssDNA promoted VSMC apoptosis and blocked the S and G2 phase in the cell cycle. Kassab and Hassan observed that the inhibition of cell proliferation caused by the inhibition of FAK activity was mainly reflected by the increase in the proportion of cells in the G2 phase and the decrease in that in the S phase [30]. In this experiment, the cell proportion in the S and G2 phase increased, suggesting that av*β*3 ssDNA may inhibit cell proliferation through multiple targets. The Ras-PI3K/MAPK signaling pathway plays an important role in the growth, proliferation, and migration of VSMCs [10, 32]. Inactivation of Ras-PI3K/MAPK signaling can inhibit cell proliferation and promote apoptosis through the mitochondrial pathway [33]. Ras-PI3K/MAPK is a downstream signaling pathway mediated by insulin receptors [34]. Studies have shown that MAPKs activated by insulin-like growth factor receptor are key regulators of cell differentiation. MAPK signaling is closely related to cell growth and proliferation [35]. Lu et al. showed that nesfatin-1 promoted the proliferation and migration of VSMCs by enhancing the activity of PI3K/AKT/mTOR [36]. Vaspin inhibited high glucose-induced proliferation and migration of VSMCs induced by inhibiting MAPK and PI3K/AKT signaling [37]. Lv et al. found that SM22*α* inhibited lamellipodium formation and migration via Ras-Arp2/3 signaling in synthetic VSMCs [38]. We showed that av*β*3 ssDNA significantly reduced the expression of Ras, p-PI3K, and p-MAPK, suggesting that av*β*3 ssDNA may exert its inhibitory function on cell proliferation and migration by affecting the Ras-PI3K-MAPK signaling pathway.

In conclusion, our study demonstrated that av*β*3 ssDNA attenuated cell proliferation and migration and promoted cell apoptosis, and the mechanism may be related to the Ras-PI3K/MAPK pathway. Thus, we suggest that av*β*3 ssDNA could be a potentially effective agent for the treatment of post-PTCA restenosis.

## Figures and Tables

**Table 1:** PCR reaction system.

Component	Volume
ssDNA (1 *μ*M)	1 *μ*L
av*β*3-F (10 *μ*M)	1 *μ*L
av*β*3-R (10 *μ*M)	1 *μ*L
2x PCR mix	10 *μ*L
ddH_2_O (DNase-free)	To 20 *μ*L
Total volume	20 *μ*L

**Table 2:** Primer sequences.

Primer	Sequence (5′-3′)
OPN-F	GCTTGGCTTACGGACTGA
OPN-R	GCAACTGGGATGACCTTG
FAK-F	TGCCATCAATACCAAAGTT
FAK-R	TCCAATACAGCGTCCAAGT
Ras-F	AAACATCAGCCAAGACC
Ras-R	TAGAAGGCATCCTCCAC
MAPK-F	AGCATTACCTTGACCAGC
MAPK-R	TTCCACGGCACCTTATTT
PI3K-F	TGTAAAACCGTCGTAAGC
PI3K-R	AACAGCAAAACATAATCG
GAPDH-F	CAAGTTCAACGGCACAG
GAPDH-R	CCAGTAGACTCCACGACAT

**Table 3:** Binding rate of each round of aptamers to positive cells.

Rounds	1	2	3	4	5	6	7	8	9	10	11	12
Binding rate (%)	7.3	12.2	18.1	24.3	29.8	34.6	39.0	43.0	44.7	45.1	45.5	45.0

**Table 4:** Affinity rate of each round of aptamers to positive cells.

Rounds	1	2	3	4	5	6	7	8	9	10	11	12
OD	0.34	0.48	0.59	0.64	0.77	0.92	1.10	1.20	1.28	1.31	1.32	1.31

## Data Availability

The data that support the findings of this study are available from the corresponding author upon reasonable request.

## Supplementary Materials

Supplementary Materials Secondary structure of the remaining four adapters.
