# Supplementary figures and images for: Avβ3 Single-Stranded DNA Aptamer Attenuates Vascular Smooth Muscle Cell Proliferation and Migration via Ras-PI3K/MAPK Pathway
Source: Cardiovasc Ther. 2020 Jan 16;2020:6869856. doi: 10.1155/2020/6869856 (PMC6995496; doi:10.1155/2020/6869856)

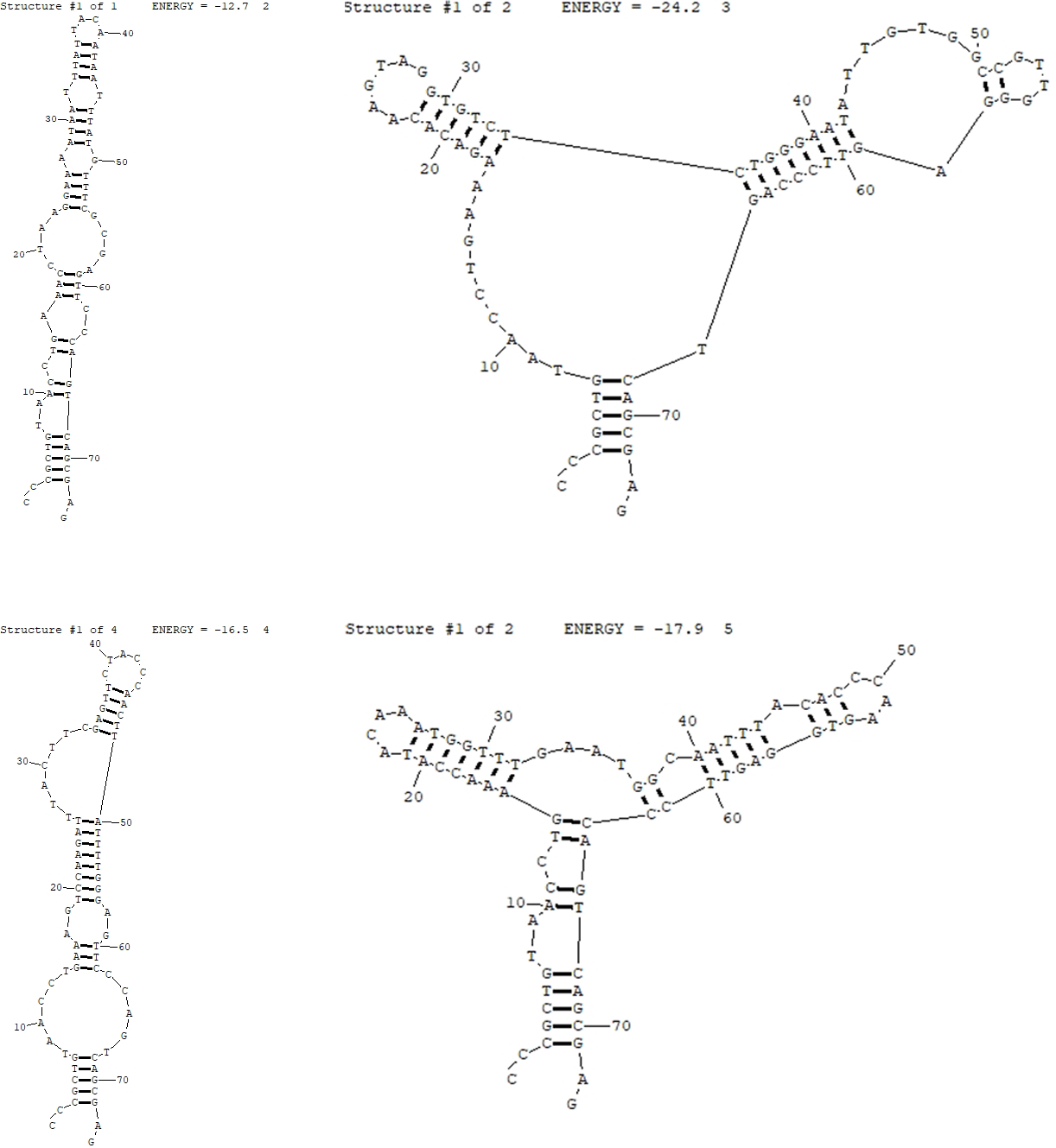


Fig. S1. Secondary structure of the remaining four adapters

Supplement: Supplementary Materials — Secondary structure of the remaining four adapters. [file 6869856.f1.docx]
